# Translation and Adaptation of the SCOFF Questionnaire to the Greek Language (G-SCOFF) Using a Tertiary-Setting Adolescent Sample

**DOI:** 10.3390/nu17020347

**Published:** 2025-01-19

**Authors:** Eleni G. Paschalidou, Athina Markopoulou, Maria G. Grammatikopoulou, Aristea Gioxari, Alexandra Foscolou, Eirini Karagiannopoulou, Tonia Vassilakou, Dimitrios G. Goulis, Kyriaki Tsiroukidou, Efstratia Daskalou

**Affiliations:** 1 Pediatric Endocrinology Unit, 3rd Department of Pediatrics, Hippokration General Hospital of Thessaloniki, Aristotle University of Thessaloniki, GR-54124 Thessaloniki, Greece; elenipasxalidou99@gmail.com (E.G.P.); eirinikar19@hotmail.com (E.K.);; 2 Department of Nutritional Sciences and Dietetics, Faculty of Health Sciences, Alexander Campus, International Hellenic University, Sindos, GR-57400 Thessaloniki, Greece; dnd4943@nutr.teithe.gr; 3 Unit of Immunonutrition & Clinical Nutrition, Department of Rheumatology and Clinical Immunology, University General Hospital of Larissa, Faculty of Medicine, School of Health Sciences, University of Thessaly, Biopolis Campus, GR-42110 Larissa, Greece; 4 Unit of Reproductive Endocrinology, 1st Department of Obstetrics and Gynecology, Faculty of Health Sciences, Medical School, Aristotle University of Thessaloniki, GR-54124 Thessaloniki, Greece; dgg@auth.gr; 5 Department of Nutritional Science and Dietetics, School of Health Sciences, University of the Peloponnese, Antikalamos, GR-24100 Kalamata, Greece; a.gioxari@go.uop.gr (A.G.);; 6 Department of Public Health Policy, School of Public Health, University of West Attica, 196 Alexandras Avenue, GR-11521 Athens, Greece; tvasilakou@uniwa.gr; 7 Department of Nutrition, General Hospital of Thessaloniki “G. Gennimatas”, 41 Ethnikis Aminis Str., GR-54635 Thessaloniki, Greece; efidask@yahoo.gr

**Keywords:** eating disorders, bulimia nervosa, binge eating disorder, rumination disorder, ARFID, nutritional assessment, undernutrition, nutritional risk

## Abstract

Background/Objectives: Feeding and eating disorders (FEDs) constitute an important mental health problem today, especially among youngsters. The Sick, Control, One, Fat, Food (SCOFF) questionnaire was developed 25 years ago and remains the most frequently applied screening tool for FEDs among adults and youngsters. The aim of the present study was to translate and adapt the SCOFF questionnaire to the Greek language, using a tertiary-setting adolescent sample. Methods: A total of 167 adolescents (86 boys, 81 girls) admitted to the pediatric outpatient clinics of the Georgios Gennimatas and Hippokration General hospitals completed the SCOFF questionnaire. Anthropometric indices were measured and dietary intake was recorded. Results: A total of 74 adolescents (44.3% of the sample) were classified as engaging in disordered eating behaviors and as possible patients with FEDs based on the SCOFF questionnaire. No differences were noted in the dietary intake between adolescents who screened positive and those who screened negative for FEDs. The body mass index z-score and obesity status were greater among children screening positive for FEDs compared to those screening negative (*p* < 0.01). One patient who was later diagnosed with anorexia nervosa was detected as a possible FED case by the questionnaire. Conclusions: The present study revealed that more than two out of five adolescents attending a pediatric clinic exhibited disordered eating behavior. The use of the G-SCOFF questionnaire is feasible and effective for FED triage in the Greek tertiary pediatric setting.

## 1. Introduction

Feeding and eating disorders (FEDs) consist of serious, debilitating complex diseases constituting an important mental health problem today, and represent a pivotal public health concern, especially among youngsters [1]. Recent data indicate that the prevalence of FEDs in Greece reaches 25% (1 in 4) of those seeking dietary recommendations from a registered dietitian nutritionist (RDN) [2]. The COVID-19 pandemic and lockdown have further augmented the prevalence of FEDs recently [3]. FEDs are related to abnormal eating and weight control behaviors, and may greatly impair quality of life, growth, and psychological and physical health [4]. FEDs can increase healthcare costs and disrupt the psychosocial functioning of both patients and their families [5,6,7,8]. Furthermore, they are associated with elevated morbidity and mortality rates [9,10]. According to Tanner [11], children and adolescents with restrictive FEDs in particular may experience exceptional medical complications due to their high energy requirements attributed to puberty onset, rapid development, and growth. Hence, in this population, the risk of irreversible complications including impeding growth and impairing brain and bone health is apparent [11]. According to the latest edition of the Diagnostic and Statistical Manual for Mental Disorders (DSM-V) [12] published by the American Psychiatric Association (APA), the FEDs umbrella encompasses anorexia nervosa (AN), bulimia nervosa (BN), binge eating disorder, other specified feeding and eating disorders (OSFEDs), unspecified feeding or eating disorders (USFEDs), pica, rumination disorder, and avoidant/restrictive food intake disorder (ARFID).

Adolescence is considered as one of the most vulnerable life stages for the development of FEDs due to the dramatic body shape transitions that occur during this period, which in turn affect body image and self-perception. A female predominance is apparent in most FEDs, with a reported 10% of adolescent females in the United States being affected by FEDs [6]. Caucasian populations tend to exhibit greater rates of AN, whereas BN appears to be more prevalent among Black, Asian, and Hispanic/Latino individuals [13,14]. With regard to sex, transgender adolescents self-report higher rates of FED diagnoses [14,15]. During the past few decades, however, FEDs have displayed an growing prevalence among adolescents [16,17,18,19] and an increasing rate among boys, affecting youngsters of all ethnicities, sizes, and socio-economic backgrounds [20].

Research has identified a hereditary aspect [21,22], triggered by a variety of environmental factors, in the development of FEDs. Social media use, sports participation, and a chronic disease diagnosis affecting body image and/or dietary intake also increase the risk of developing FEDs [23,24,25,26]. In parallel, childhood trauma, other mental health diagnoses, bad family relationships, social pressure to meet body weight and appearance standards, and perfectionism further multiply the risk for FEDs [27,28,29], adding more parameters to its diverse etiology.

It should be noted; however, that in healthcare settings, clinicians often face difficulties in identifying FEDs, OSFEDs, or even simple disordered eating behaviors [30]. This is further reinforced by the inadequate and inhomogeneous screening procedures, which are often exacerbated by personal, time-restriction, knowledge-related, and health-system-related barriers [30]. Although clinicians report awareness of some FED diagnostic criteria, they appear to be less savvy regarding some FED and OSFED diagnoses [30]. Nonetheless, according to the American Academy of Pediatrics, pediatricians should screen for FEDs in annual health supervisions or sports examinations, using longitudinal body weight and stature data, as well as signs of disordered eating [31,32]. In parallel, the American Academy of Child and Adolescent Psychiatry [33] recommends screening every preteen and adolescent patient for possible FEDs using weight and stature assessments, as well as screening questions regarding dietary patterns and body image.

Although the extent of the problem is not a matter of dispute, less than half of the screening forms used by pediatricians include questions specific to FEDs, with many barriers being identified towards prompt FED recognition and diagnosis [34]. The Sick, Control, One, Fat, Food (SCOFF) questionnaire [35] was developed approximately 25 years ago and still constitutes the most frequently used screening tool for FED triage among adults and youngsters to date [36]. The aim of the present study was to translate and adapt the SCOFF questionnaire to the Greek language, using a tertiary-setting adolescent sample.

## 2. Materials and Methods

### 2.1. Sample Recruitment

Adolescent patients who visited the pediatric outpatient clinic of the Georgios Gennimatas General Hospital and the Pediatric Endocrinology Unit situated at Hippokration General Hospital, both in Thessaloniki, were recruited between October 2022 and May 2024. Inclusion criteria included (i) adolescents aged between 10 and 16 years old, (ii) admitted to outpatient pediatric clinics, (iii) who were willing to participate, (iv) with parental/guardian consent, (v) with any diagnosis or admission etiology, (vi) and who were able to communicate in the Greek language adequately to understand the questions and reply effortlessly. A total of 167 adolescents (86 boys, 81 girls), with a median age of 11.7 (3.2) years, fulfilled the criteria and were included in the sample.

The medical diagnoses varied among the participating children, ranging from metabolic issues to hormonal and growth problems, gastrointestinal problems, eye and vision issues, infectious conditions, cardiovascular and hematological issues, and nutritional and autoimmune diseases. The characteristics of the sample are presented in Table 1.

#### 2.1.1. Ethical Permission

Permission for this study was granted by the Director of the 3rd Health Prefecture of Macedonia (Δ3β/8918, 4 April 2023), as well as by the Hippokration Hospital Scientific Committee (307/29-04-2024). Furthermore, informed consent was provided by all parents/guardians of the participating children.

#### 2.1.2. Anthropometry

The body weight and stature of participants were measured to the nearest gram and centimeter, respectively, by experienced dietitians (A.M., E.P. and E.K.) in the morning, with the subjects being barefoot, while wearing the minimum amount of clothes possible. A mechanical scale (Seca 700, Seca, Hamburg, Germany) and a wall-mounted stadiometer (Harpenden, Holtain, Crymych, UK) were used.

The body mass index (BMI) was calculated for all participants as the ratio of body weight (kg), divided by stature (m^2^). The World Health Organization’s (WHO) Anthro software v.3.2 (WHO, Geneva, Switzerland) [37] for the assessment of growth and development of children and adolescents, was used to calculate the BMI z-score (BMIz), weight-for-age z-score (WAZ), and height-for-age z-score (HAZ). The Anthro software is based on the WHO child growth standards and growth curves [38,39]. Weight status was defined according to the WHO as underweight when BMIz ≤ −1.00, normoweight when BMIz ranged between −0.99 and 0.99, overweight when BMIz ≥ 1.00, and obesity when BMIz ≥ 2.00.

The triceps skinfold was measured (Harpenden, Holtain, Crymych, UK) by two experienced dietitians (E.D. and E.P.) and recorded for all participants, as a proxy for nutritional status. Middle upper arm circumference (MUAC) was measured on a straight left arm, midway between the tip of the shoulder and that of the elbow, using a common anelastic tape, according to the WHO guidelines [40].

### 2.2. The SCOFF Questionnaire

For disordered eating triage, the SCOFF questionnaire was employed. The SCOFF questionnaire uses five questions to address core features of AN and BN [35]. The name of the questionnaire consists of an acronym based on the domains assessed in each of the five questions, namely Sick, Control, One Stone, Fat, and Food. For the scoring, one point is given to each positive answer. A cumulative score of 2 or greater denotes suspicion of an existing FED diagnosis (AN or BN), indicating a possible FED case (tool sensitivity: 100%; specificity: 87.5%) [35,36] and adherence to disordered eating behaviors.

Various studies have used and validated this questionnaire in adolescent populations [41,42,43]. The SCOFF questionnaire has been shown to be highly effective in screening for possible FEDs [35], simple and easy to use, memorable, and very easy to score [35].

### 2.3. Translation and Adaptation of the SCOFF Questionnaire

Permission for the translation of the questionnaire was kindly granted by its main author, Professor John F. Morgan, *MA*, *MD*, *FRCPsych*, after personal communication. The four-step forward–backward method [44] was applied for the tool’s translation, which involved a total of four bilingual experts (M.G.G., E.D., A.G., and A.F.).

### 2.4. Dietary Intake

Previous 24 h diet recalls were collected in detail from all participants by an experienced dietitian (A.M.), with the help of their parents/guardian. The use of food photos with realistic sizes facilitated this process. Records were analyzed using the Cronometer software (Web-version, Cronometer Software Inc., Vancouver, BC, Canada) [45].

### 2.5. Statistical Analyses

Statistical analyses were performed using Jamovi (Version 2.3.21.0). The normality of data was graphically explored with Q–Q plots. Continuous variables were presented as median plus interquartile range (IQR). Categorical values were expressed as counts (n). The Kuder–Richardson (K-R) formula [46] was used for the assessment of internal consistency reliability of the SCOFF questionnaire. The K-R formula is used for measures with dichotomous choices, like the SCOFF questionnaire. Values range between 0 and 1, with higher values indicating greater validity.

### 2.6. Sample Size Calculation

We performed sample size calculation and we hypothesized a percentage frequency of the outcome factor in the population of 50% (±5), within confidence levels of 80% and a design effect of 1 (randomized sample). As a result, the minimum sample size was estimated to be 165.

## 3. Results

### 3.1. Translation of the SCOFF Questionnaire

**Figure 1:**
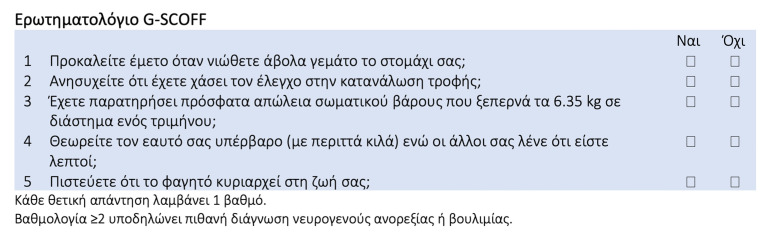
Translated SCOFF questionnaire in the Greek language (G-SCOFF) [35]. SCOFF, Sick, Control, One stone, Fat, Food.

The translated version of the Greek SCOFF (G-SCOFF) questionnaire is presented in Figure 1.

### 3.2. Results of the SCOFF Questionnaire

SCOFF questionnaire data are presented in Table 2. A total of 74 adolescents (44.3% of the sample), namely 33 boys and 41 girls, were classified as possible patients with FEDs, according to the SCOFF questionnaire (total score ≥ 2). No differences were noted in the age of participants identified with possible FEDs versus the rest of the sample.

One patient that was later diagnosed with AN was detected as a possible FED case by the questionnaire. Nearly 7 out of 10 (69.4%) of participants with obesity demonstrated disordered eating behaviors (SCOFF total score ≥ 2).

### 3.3. Differences in Anthropometry and Dietary Intake

Differences in anthropometry among adolescents identified to be engaging in disordered eating behaviors (SCOFF total score ≥ 2) compared to the rest of the sample are presented in Table 3. Significant differences were observed only for BMIz and obesity status (*p* < 0.01 for both).

The dietary intakes of adolescents identified as having disordered eating behaviors (SCOFF total score ≥ 2) compared to their FED-free counterparts are presented in Table 4. No differences were detected between the groups. In parallel, no difference was noted regarding the % of energy intake meeting energy requirements, between adolescents adhering to disordered eating practices, versus the rest.

### 3.4. Internal Consistency and Validity of the Translated SCOFF Questionnaire

Reliability and validity analyses are presented in Table 5. The K-R coefficient of the SCOFF questionnaire was calculated at 0.354, indicating low internal consistency for all items in the SCOFF questionnaire. Spearman’s correlation coefficient analysis indicated that only items E1, E2, E4, and E5 showed validity.

## 4. Discussion

The present study aimed to translate the SCOFF questionnaire into the Greek language and apply it to a pediatric population. Despite its low validity, the questionnaire was able to detect one patient who was diagnosed with AN. Additionally, the results revealed that 44.3% of children and adolescents attending tertiary pediatric clinics screen positive for disordered eating behaviors. Apart from BMIz and obesity status, no other differences were noted in the anthropometry, growth, or nutrient intake between children and adolescents who were identified as possible FED cases and those who were not.

The present study revealed that approximately half (44.3%) of the adolescents attending the pediatric clinic exhibit disordered eating behavior. In type 1 diabetes clinics, the prevalence of disordered eating behavior has been shown to exceed 20% [47]. Outside the clinical setting, one-fifth of the children and adolescent population have been shown to engage in disordered eating after screening positive using the SCOFF questionnaire [19]. Overall, research is unanimous on the greater risk for developing disordered eating behaviors among adolescents with chronic illness compared to their healthy peers [24,48]. Apart from the healthcare environment and the general population, the SCOFF questionnaire has also been used in the school setting, showing efficacy in uncovering hidden FEDs among students [49,50,51]. It is important to note, however, that disordered eating and FEDs are two different situations, since not all children who engage in disordered eating behaviors are eventually diagnosed with an FED [19,52].

In the present study, the SCOFF tool managed to identify one case of AN that was included among the participants. In previous studies, the tool was efficient at detecting confirmed FED cases among adult women in the primary care setting [53], as well as among French women with FEDs, compared to healthy controls [54]. The questionnaire was also shown to have acceptable reliability in a sample of Danish adolescents attending a specialized eating disorder clinic [55]. Clinical algorithms have been suggested as secondary tools to aid the use of the SCOFF questionnaire, serving to classify positive cases into four broad FED categories, including restrictive, bulimic, or hyperphagic disorder [56].

Since the present sample consisted of a tertiary pediatric population, it is possible that the lack of differences observed in the anthropometry and dietary intake of participants may be due to their underlying diagnoses. Since the primary aim of this study was to translate and use the SCOFF tool in a Greek adolescent sample, no restrictions were posed regarding the inclusion criteria. In parallel, we aimed for this to be a pragmatic study, performed in the real-world pediatric clinic setting, and thus the results may well be valid for this site. Furthermore, previous research has suggested that in extreme heavy or small somatotypes, the anthropometry often induces biases towards the detection of FEDs [57]. In children and adolescents, where the possible observed range in body weight is much smaller compared to adults, it may be difficult to detect anthropometric differences between those demonstrating disordered eating behaviors and those who are not. Furthermore, disordered eating behaviors are equally presented by children with obesity and overweight as among children with underweight [58,59,60,61].

Similarly, energy and nutrient intake values are generally lower in children and adolescents compared to adults, and thus larger samples are required to detect differences. Also, just like with anthropometry, the underlying diagnoses of the children may well mask disordered dietary intakes, hampering the ability to detect differences in this sample. It should also be noted, however, that disordered eating does not always coincide with a reduced dietary intake [62,63,64], especially given the fact that many children and adolescents engaging in disordered eating are, in fact, obese [58,59,60].

According to a recent diagnostic test accuracy (DTA) meta-analysis [36], although the SCOFF questionnaire consists of a simple and useful triage tool for AN and BN in particular, the evidence supporting its use for screening for the whole spectrum of DSM-V FEDs (including ARFID, binge eating disorder, pica, or OSFEDs) in the primary care and community-based settings remains limited. As a result, authors have raised concerns regarding the generalizability and reliability of the SCOFF questionnaire for other FED diagnoses [36]. A recent DTA meta-analysis showed that the pooled validity of the tool is high across samples, with a sensitivity of 0.86 and a specificity of 0.83 [36]. According to an older DTA [65], when the tool was used within structured interviews, its efficacy was significantly improved, reaching a sensitivity is 0.88 and a specificity of 0.925. For this reason, the SCOFF questionnaire has been included in the US Preventive Services Task Force (USPSTF) [66] recommended tools for FED screening. An alternative tool is the MEBS (Minnesota Eating Behavior Survey) [67], which can be applied to both adolescents (from 10 years of age) and adults.

FEDs impose a significant economic healthcare cost to the community [68] and a high clinical burden [69]. Research in other countries [70,71] has showed that the SCOFF questionnaire can be easily applied in the hospital setting and has the ability to detect possible cases and exclude non-cases of most commonly occurring FEDs. The COVID-19 pandemic in particular has increased the risk for emergency department visits and hospitalization by 66% and 37%, respectively [72]. Furthermore, elevated rates of disordered eating are observed in sexual minority youth [73,74], although no such cases were observed herein.

After completion of a positive screening for FEDs, the pediatrician must arrange for the delivery of appropriate care [75]. Adolescents with mild nutritional, medical, or psychological issues can be managed in collaboration with outpatient dietitians and mental health professionals with FED-specific experience and expertise [75]. For adolescents with more health complications and issues, the collaboration of multidisciplinary teams has been recommended, including a pediatrician, dietitian, psychiatrist, psychologist, psychiatric nurse, social worker, community support, and other medical specialists (e.g., gastroenterologist, endocrinologist), in order to address and manage disordered eating [76]. As noted by several scientific societies, healthcare practitioners should be able to recognize signs of FEDs, including preoccupation with food, body weight, appearance, calories, and engaging in weight-control behaviors such as fasting, skipping meals, and vomiting to lose weight [6,66,77,78]. Unfortunately, however, most have minimal training in FEDs, and thus face difficulties in identifying, diagnosing and eventually managing such conditions [30,79,80]. For this, healthcare providers—including pediatricians—should routinely enquire about eating habits, as a component of the overall health assessment [1]. Furthermore, given the vulnerability of adolescents, providers caring for these patients should know how to identify, approach, and manage these age-related complications [11]. Given that a shortage of time and a lack of staff confidence around the use of FED screening tools have been suggested as possible barriers to routinely screen patients, educating healthcare professionals should be of high priority [47].

The limitations of the present research include the cross-sectional nature of the study. The sample size was sufficient given that the SCOFF questionnaire has five items and according to the rule of thumb, at least 10 participants are required for each domain within a questionnaire in order to produce robust validation results [81]. Finally, one should not overestimate SCOFF questionnaire’s ability to detect FEDs, as it was designed for the detection of AN and BN [35], prior to the publication of the DSM-V [12]. This means that it does not screen for other FEDs, including ARFID, or atypical AN, entities that were included in the FED pool with the introduction of the DSM-V [12].

Future research should apply the SCOFF questionnaire in larger pediatric care samples and conduct interventions to educate healthcare professionals on the proper screening and management of disordered eating and FEDs among adolescents. Furthermore, the SCOFF questionnaire should also be validated for other FEDs asides from AN and BN.

## 5. Conclusions

The present study showed that the SCOFF tool can easily be used in the Greek pediatric tertiary setting, while in parallel, it is also able to detect FED cases. Furthermore, it showed that screening for possible FEDs is important in routine clinical practice, as a great proportion of adolescents exhibit disordered eating behaviors, irrespective of their weight status.

## Figures and Tables

**Table 1:** Characteristics of the participating adolescents (N = 167).

Boys/Girls (*n*)	86/81
Age (years)	11.7 (3.2)
Body weight (kg)	44 (27)
Stature (cm)	147 (18.5)
BMIz	0.77 (1.83)
Weight status normoweight/overweight/obese (*n*)	88/33/36

BMIz, body mass index z-score; IQR, interquartile range; Presented as counts (*n*) or as means and standard deviations for normally distributed variables or medians with their respective IQR for non-normal distributed variables (body weight and BMIz).

**Table 2:** Results of the SCOFF [35] questionnaire (N = 167) *.

		Yes (*n*)	No (*n*)	*p* Value
**S**	Do you make yourself **Sick** because you feel uncomfortably full?	42	125	<0.001
**C**	Do you worry you have lost **Control** over how much you eat?	52	115	<0.001
**O**	Have you recently lost more than **One stone** (6.35 kg) in a three-month period?	1	166	<0.001
**F**	Do you believe yourself to be **Fat** when others say you are too thin?	43	124	<0.001
**F**	Would you say **Food** dominates your life?	119	48	<0.001

* Results are presented as counts (*n*). For the comparison of proportions, the chi-square test was used. The level of statistical significance was set below 0.05. SCOFF, Sick, Control, One stone, Fat, Food [35].

**Table 3:** Anthropometric evaluation of participating children and adolescents according to SCOFF score (N = 167) *.

	Possible FEDs ^†^ (*n* = 74)	FED-Free ^‡^ (*n* = 93)	Total (N = 167)	*p* Value
MUAC (cm)	90 (37.5)	90 (56.3)	90 (45)	NS
TSF (mm)	62.5 (43.3)	90 (45)	75 (43.5)	NS
BMIz	1.03 (2.38)	0.65 (1.64)	0.77 (1.83)	0.008
WAz	1.3 (2.16)	0.8 (1.66)	0.91 (1.91)	NS
HAz	0.31 (1.83)	0.4 (1.77)	0.17 (1.88)	NS
Obesity (BMIz ≥ 2.0) (*n*, *%*)	25 (33.8)	11 (11.8)	36 (21.6)	<0.001

* Continuous variables are presented as medians and their respective IQR; ^†^ SCOFF total score ≥ 2; ^‡^ SCOFF total score < 2; categorical variables are presented as counts (*n*) and frequencies. For the comparison between the two groups, the Mann–Whitney rank test was used. The level of statistical significance was set below 0.05. BMI, body mass index; BMIz, BMI-for-age z score; FED, feeding and eating disorder; HAz, height-for-age z score; IQR, interquartile range; MUAC, middle upper arm circumference; NS, not significant; SCOFF, Sick, Control, One stone, Fat, Food [35]; TSF, triceps skinfold; WAz weight-age z score.

**Table 4:** Dietary intake of participating children and adolescents according to SCOFF score (N = 167) *.

Dietary Intake	Possible FEDs (*n* = 74)	FED-Free (*n* = 93)	Total (N = 167)	*p* Value
Energy (kcal/d)	1553 (713)	1453 (883)	1498 (802)	NS
Total carbohydrates (g/d)	182.4 (91)	170.2 (102.2)	172 (100.1)	NS
Fibers (g/d)	12.2 (11.2)	12.8 (8.95)	12.5 (10.2)	NS
Sugars (g/d)	51.1 (37.4)	53.5 (48.4)	53.3 (42.5)	NS
Total fats (g/d)	66.4 (45.5)	59.1 (50.1)	63.8 (48.6)	NS
MUFA (g/d)	19.7 (21.1)	21.0 (21.2)	20 (21.7)	NS
PUFA (g/d)	8.7 (9.7)	8.7 (8.9)	8.7 (9.0)	NS
n-3 fatty acids (g/d)	1.0 (0.9)	0.9 (1.2)	1.0 (1.0)	NS
SFA (g/d)	22.4 (18.4)	20.0 (15.1)	21.2 (16.6)	NS
Trans fatty acids (g/d)	0.8 (0.9)	0.9 (0.9)	0.8 (0.9)	NS
Cholesterol (g/d)	171.6 (227.7)	174 (182.7)	174 (214)	NS
Total proteins	67.3 (40.6)	56.6 (42.0)	60.9 (41.2)	NS
Isoleucine (g/d)	2.2 (1.9)	2.0 (1.7)	2.1 (1.9)	NS
Leucine (g/d)	4.0 (3.4)	3.5 (2.6)	3.7 (3.0)	NS
Valine (g/d)	2.7 (2.1)	2.5 (1.8)	2.6 (2.0)	NS
Vitamin B12 (μg/d)	2.7 (2.7)	2.6 (2.4)	2.6 (2.7)	NS
Folic acid (μg/d)	345 (278)	349 (252)	348 (260)	NS
Vitamin A (μg/d)	302 (280)	275 (292)	285 (301)	NS
Vitamin C (mg/d)	28.1 (48.9)	30 (59)	29.3 (56)	NS
Vitamin D (IU/d)	124.4 (161)	117.1 (160.5)	123 (160)	NS
Vitamin E (mg/d)	5.65 (5.9)	5.4 (6.3)	5.6 (6.1)	NS
Ca (mg/d)	712 (521)	693 (575)	704 (540)	NS
Fe (mg/d)	10.2 (6.7)	9.4 (7.6)	9.7 (7.0)	NS
Zn (mg/d)	7.2 (5.9)	6.0 (4.4)	6.6 (4.9)	NS

* Continuous variables are presented as median plus interquartile range (IQR). For the comparison between the two groups, the Mann–Whitney rank test was used. The level of statistical significance was set below 0.05. Ca, calcium; Fe, Iron; FED, meeding and eating disorders; MUFA, monounsaturated fatty acids; NS, not significant; PUFA, polyunsaturated fatty acids; SCOFF, Sick, Control, One stone, Fat, Food [35]; SFA, saturated fatty acids; Zn, zinc.

**Table 5:** Internal consistency and validity of the G-SCOFF questionnaire (N = 167) *.

		Internal Consistency K–R (if Item Deleted)	Validity Spearman’s Correlation Coefficient	
Q1	S	0.172	0.634	*p* < 0.001
Q2	C	0.132	0.693	*p* < 0.001
Q3	O	0.375	0.107	NS
Q4	F	0.370	0.473	*p* < 0.001
Q5	F	0.393	0.506	*p* < 0.001

* Level of statistical significance was set at 0.05. K–R, Kuder–Richardson formula [46]; NS, not significant; Q, question; G-SCOFF, Greek version of the Sick, Control, One stone, Fat, Food [35].

## Data Availability

The original contributions presented in the study are included in the article, further inquiries can be directed to the corresponding author.

## Abbreviations

The following abbreviations are used in this manuscript:

AN	anorexia nervosa
APA	American Psychiatric Association
ARFID	avoidant/restrictive food intake disorder
BMI	body mass index
BMIz	body mass index z-score
BN	bulimia nervosa
DSM	Diagnostic and Statistical Manual for Mental Disorders
DTA	diagnostic test accuracy
FEDs	feeding and eating disorders
HAz	height-for-age z-score
IQR	interquartile range
K-R	Kuder–Richardson
MEBS	Minnesota Eating Behavior Survey
MUAC	middle upper arm circumference
MUFA	monounsaturated fatty acids
OSFEDs	other specified feeding and eating disorders
PUFA	polyunsaturated fatty acids
RDN	registered dietitian nutritionist
SCOFF	Sick, Control, One, Fat, Food
SFA	saturated fatty acids
TSF	triceps skinfold
USFEDs	unspecified feeding or eating disorders
WAz	weight-for-age z-score
WHO	World Health Organization
